# Supplementary figures and images for: Global transcriptional regulation of innate immunity by ATF-7 in C. elegans
Source: PLoS Genet. 2019 Feb 21;15(2):e1007830. doi: 10.1371/journal.pgen.1007830 (PMC6400416; doi:10.1371/journal.pgen.1007830)

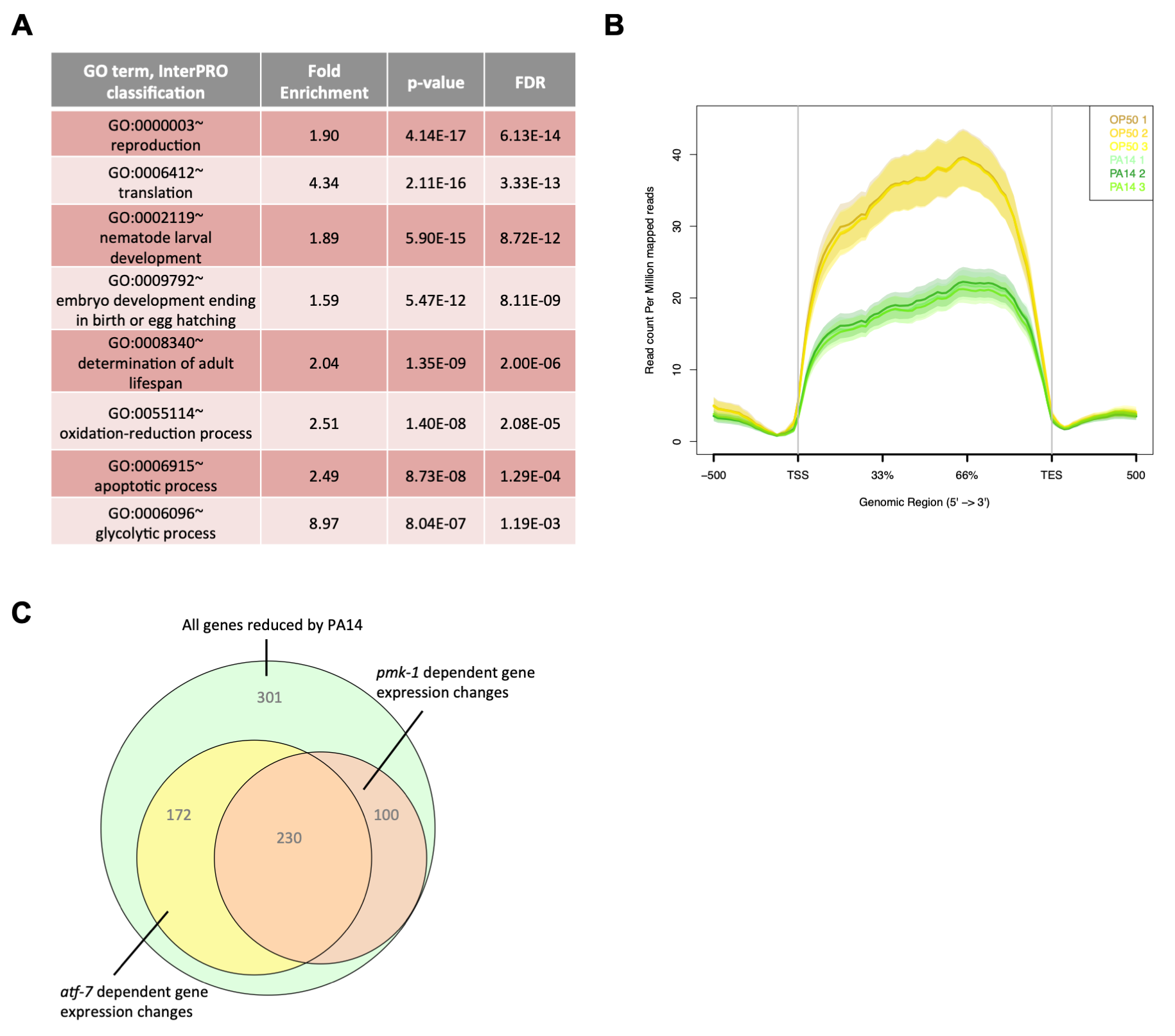

Supplement: S1 Fig — (A) Top GO terms and InterPRO classifications of transcripts that are significantly downregulated (adjusted p-value < 0.05) in N2 animals exposed to PA14 versus OP50. (B) Average expression (RPM) across the gene body of genes that are two-fold downregulated by exposure to pathogenic PA14. (C) Venn diagram of decreased genes that are dependent upon pmk-1 or atf-7 for complete (i.e. greater than two-fold, adjusted p-value < 0.05) downregulation by PA14. (TIF) [file pgen.1007830.s001.tif]

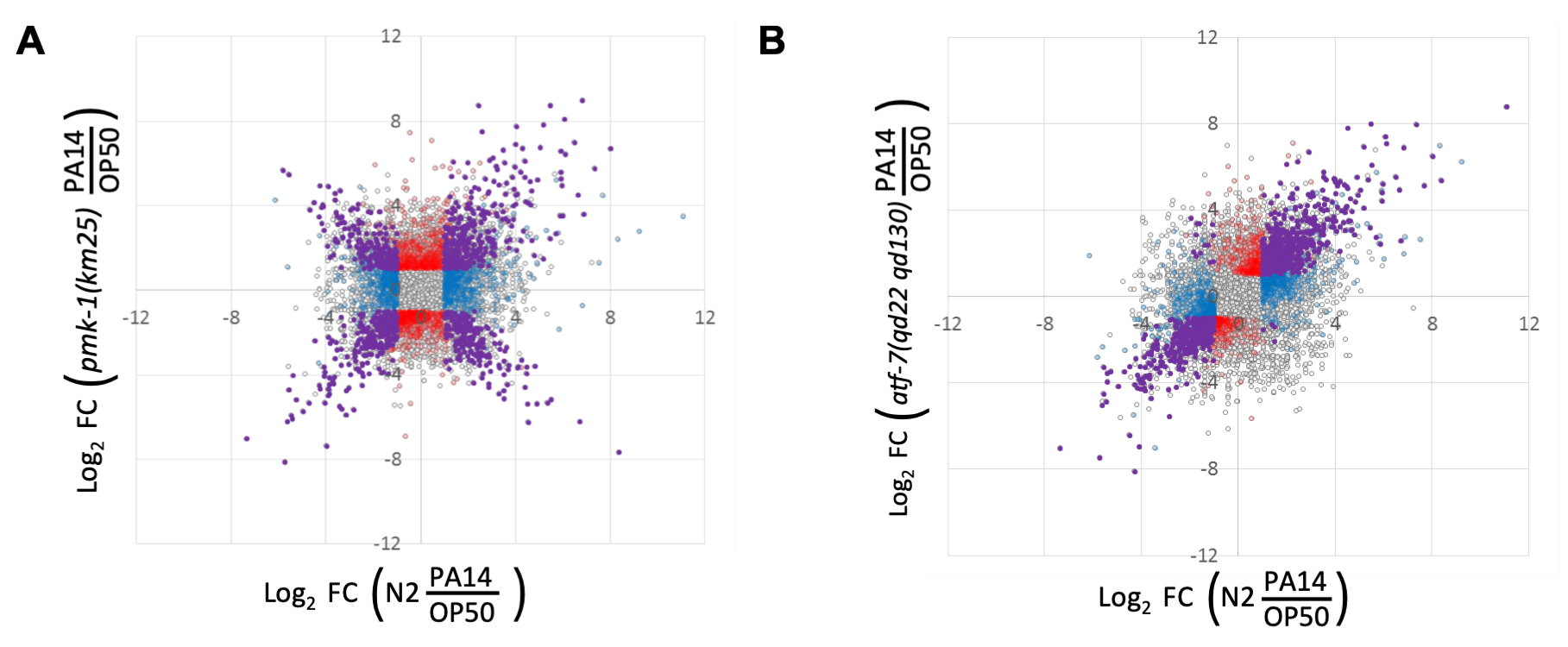

Supplement: S2 Fig — 2x2 comparison of genes differentially expressed by exposure to PA14 in N2 animals (x- axis) or upon loss of pmk-1 (A) or atf-7 (B) (y-axis). Transcripts highlighted in purple correspond to genes that were significantly different compared to OP50 in both genotypes (adjusted p-value of <0.05). Blue dots indicate genes that are significantly different in the N2 PA14/OP50 comparison only. Red dots represent genes that reach significance in only the mutant condition. Grey dots indicate genes with detected transcripts in at least one condition being compared, but that failed to reach significance cutoffs in either data set. (TIF) [file pgen.1007830.s002.tif]

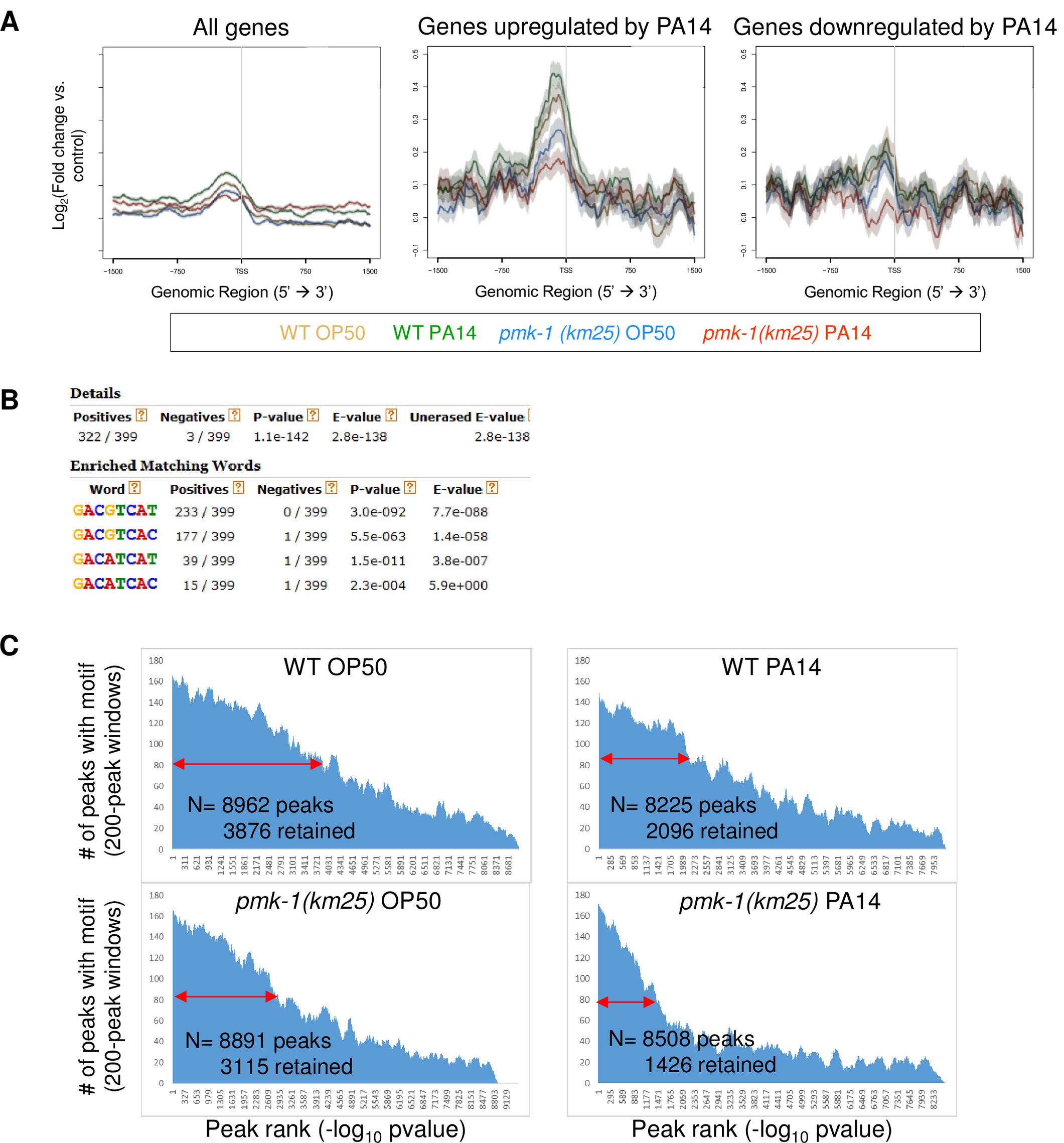

Supplement: S3 Fig — (A) Metagene analysis of ATF-7::GFP binding in all four ChIP conditions across all genes, genes that are two-fold upregulated, or two-fold downregulated by RNA-seq upon exposure to PA14 in a wild-type (N2) background. Shading represents standard error among replicates. (B) Motif analysis of ATF-7::GFP ChIP peaks. The top 400 peaks were considered for motif analysis. (C) Ranked ATF-7::GFP peaks called in animals in all four ChIP conditions. Double red arrow indicates the peaks that were retained for further analysis. (TIF) [file pgen.1007830.s003.tif]

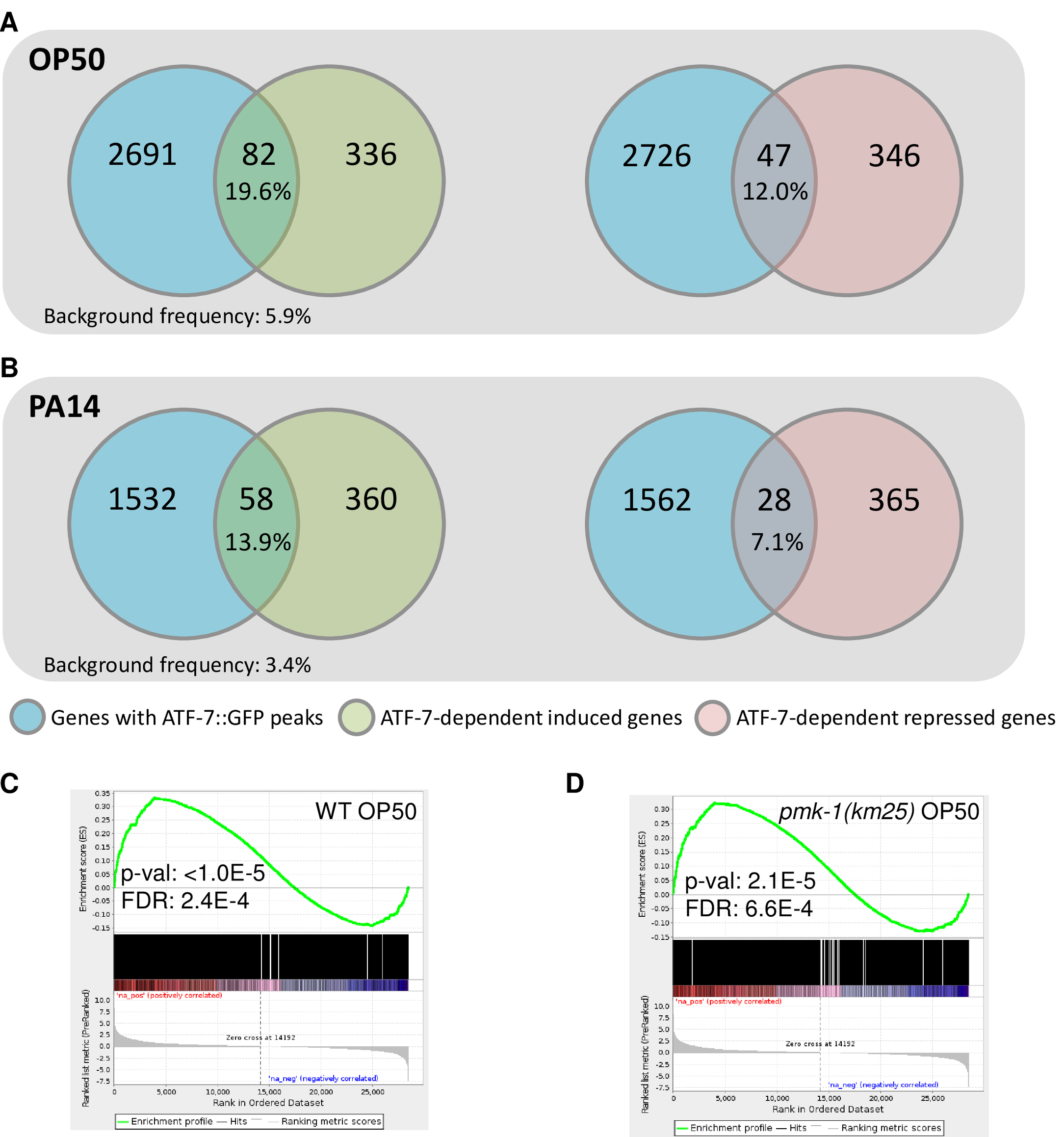

Supplement: S4 Fig — (A,B) Overlap of genes whose promoters are bound by ATF-7::GFP (blue, E. coli exposure (A), or P. aeruginosa exposure (B)) and genes whose expression is induced (green) or repressed (red) by P. aeruginosa exposure in an ATF-7-dependent manner. Percentages reflect the portion of ATF-7 dependent genes that are also bound by ATF-7. Bonferroni-adjusted hypergeometric p-value < 1E-4 for all overlaps. (C,D) Gene Set Enrichment Analysis (GSEA) of transcripts detected by RNA-seq (ranked from most upregulated to most downregulated upon PA14 exposure in N2 animals) for association with ATF-7::GFP peaks in WT (C) or pmk-1(km25) mutant (D) animals exposed to OP50. (TIF) [file pgen.1007830.s004.tif]

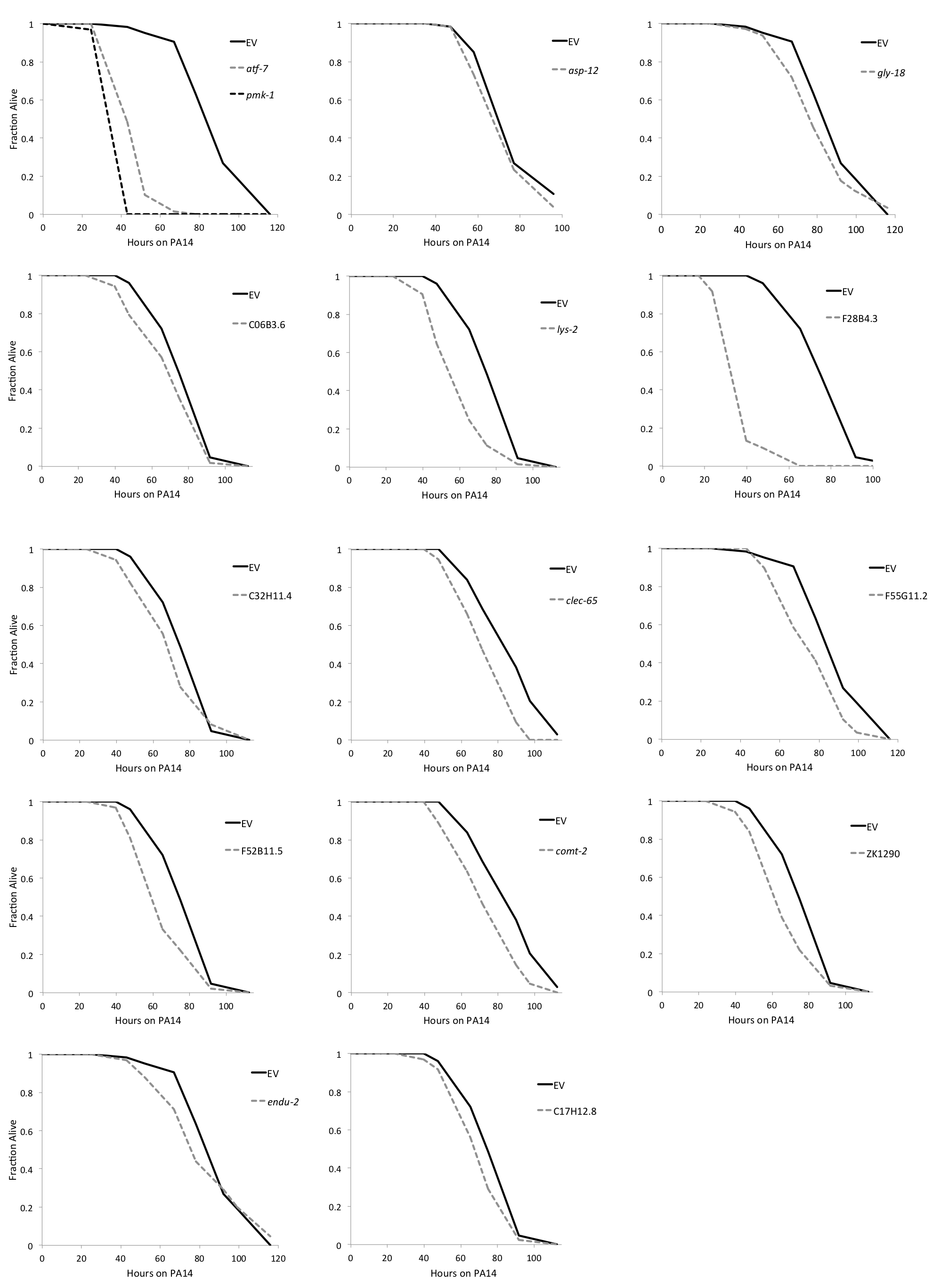

Supplement: S5 Fig — Representative survival curves of animals treated with RNAi against indicated genes that resulted in a significant (p-value < 0.05 by log-rank test) reduction in survival on PA14 compared to EV controls in 2/2 experiments. Animals were treated with RNAi for two generations prior to exposure to PA14. EV refers to HT115 carrying the Empty Vector control plasmid, L4440. (TIF) [file pgen.1007830.s005.tif]

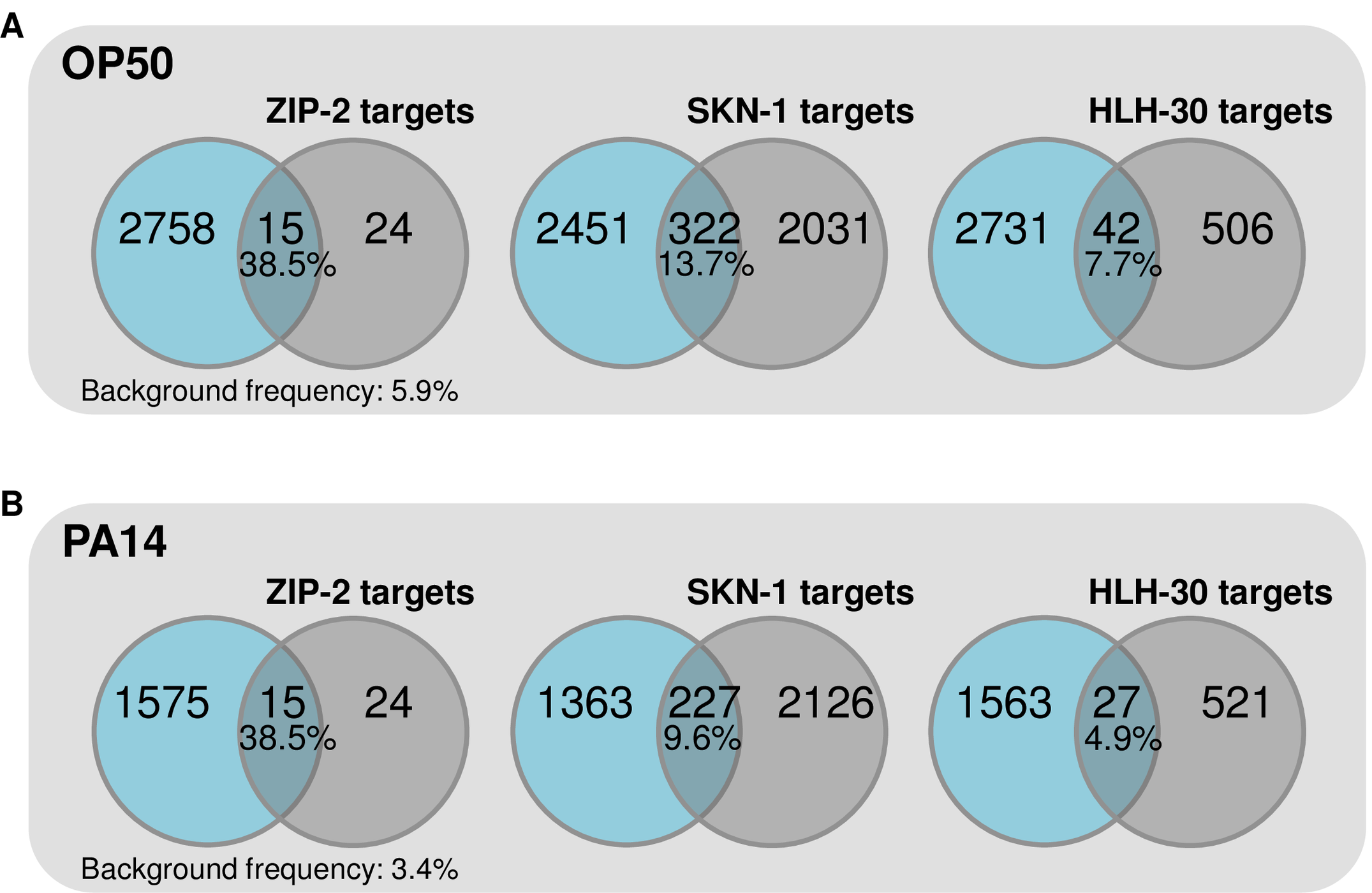

Supplement: S6 Fig — Percentages reflect the portion of ATF-7 dependent genes that are also bound by the transcription factor of interest. Target gene sets were identified using WormExp [18,38–41]. Statistical significance was assessed by hypergeometric test followed by Bonferroni correction, n.s- not significant. (TIF) [file pgen.1007830.s006.tif]

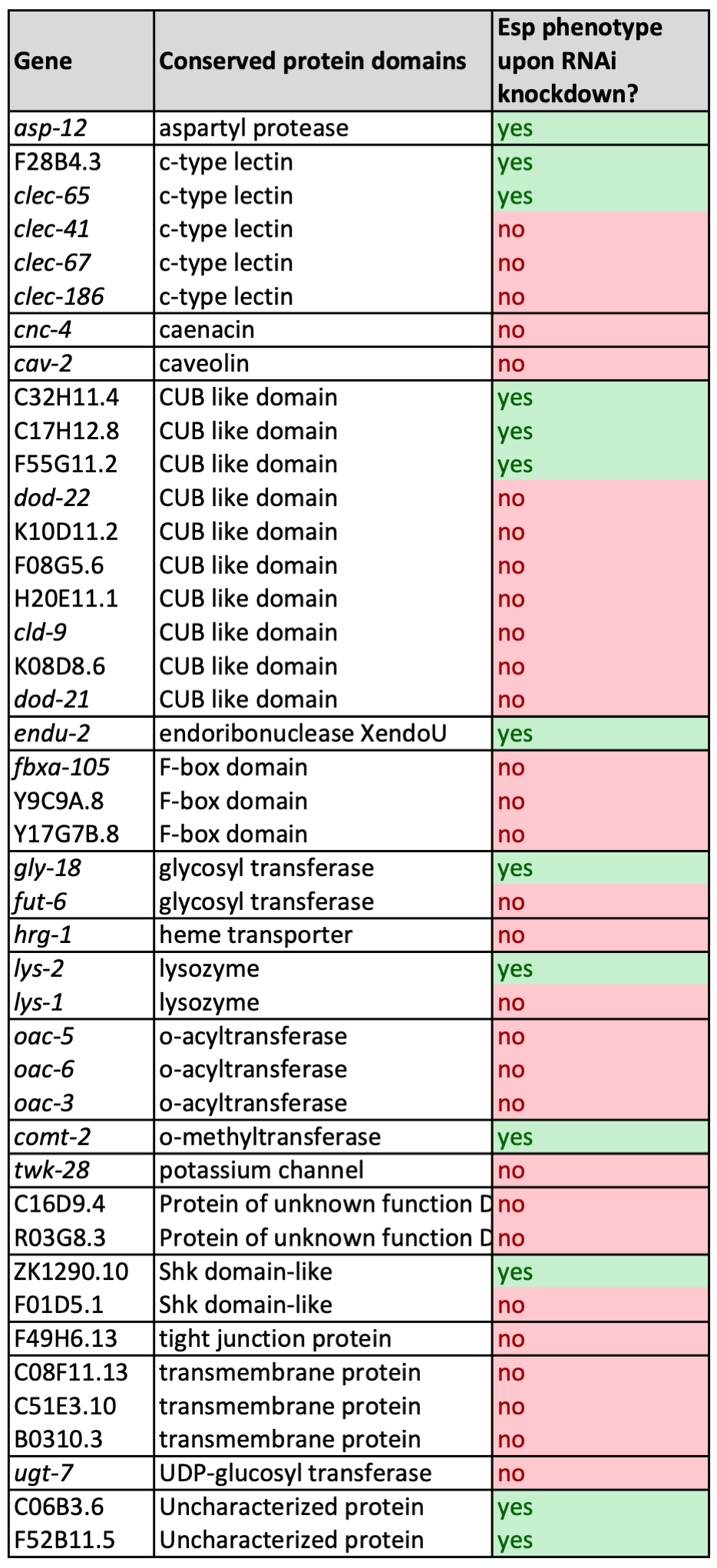

Supplement: S3 Table — Protein domains classified using the David 6.8 Functional Annotation Tool. “Yes,” indicates a significant (p-value < 0.05 by log-rank test) reduction in survival on PA14 compared to Empty Vector control in 2/2 experiments. (TIF) [file pgen.1007830.s009.tif]
